# The Stress Response Factors Yap6, Cin5, Phd1, and Skn7 Direct Targeting of the Conserved Co-Repressor Tup1-Ssn6 in *S. cerevisiae*

**DOI:** 10.1371/journal.pone.0019060

**Published:** 2011-04-28

**Authors:** Sean E. Hanlon, Jason M. Rizzo, Deirdre C. Tatomer, Jason D. Lieb, Michael J. Buck

**Affiliations:** 1 Department of Biology, Carolina Center for Genome Sciences and the Lineberger Comprehensive Cancer Center, University of North Carolina at Chapel Hill, Chapel Hill, North Carolina, United States of America; 2 Department of Biochemistry and the Center of Excellence in Bioinformatics and Life Sciences, State University of New York at Buffalo, Buffalo, New York, United States of America; Tulane University Health Sciences Center, United States of America

## Abstract

Maintaining the proper expression of the transcriptome during development or in response to a changing environment requires a delicate balance between transcriptional regulators with activating and repressing functions. The budding yeast transcriptional co-repressor Tup1-Ssn6 is a model for studying similar repressor complexes in multicellular eukaryotes. Tup1-Ssn6 does not bind DNA directly, but is directed to individual promoters by one or more DNA-binding proteins, referred to as Tup1 recruiters. This functional architecture allows the Tup1-Ssn6 to modulate the expression of genes required for the response to a variety of cellular stresses. To understand the targeting or the Tup1-Ssn6 complex, we determined the genomic distribution of Tup1 and Ssn6 by ChIP-chip. We found that most loci bound by Tup1-Ssn6 could not be explained by co-occupancy with a known recruiting cofactor and that deletion of individual known Tup1 recruiters did not significantly alter the Tup1 binding profile. These observations suggest that new Tup1 recruiting proteins remain to be discovered and that Tup1 recruitment typically depends on multiple recruiting cofactors. To identify new recruiting proteins, we computationally screened for factors with binding patterns similar to the observed Tup1-Ssn6 genomic distribution. Four top candidates, Cin5, Skn7, Phd1, and Yap6, all known to be associated with stress response gene regulation, were experimentally confirmed to physically interact with Tup1 and/or Ssn6. Incorporating these new recruitment cofactors with previously characterized cofactors now explains the majority of Tup1 targeting across the genome, and expands our understanding of the mechanism by which Tup1-Ssn6 is directed to its targets.

## Introduction

Eukaryotic enhancers often consist of adjacent binding sites for multiple proteins that work together cooperatively to recruit co-activator proteins [1]. These nucleoprotein complexes, often referred to as enhanceosomes, consist of enhancer DNA packaged into chromatin, sequence-specific activators, co-activators, and general transcription machinery [2]. Despite extensive study of enhanceosomes, there has been relatively little examination of the recruitment of co-repressors and repressor complex formation. One fairly well-characterized example is the conserved Drosophila co-repressor Groucho [2]. Groucho belongs to the Transducin-like Enhancer of split (TLE) family of repressors [3] and has been shown to be recruited synergistically by the Drosophila proteins Dead ringer, Dorsal, and Capicua to repress transcription [4], [5].

In budding yeast, Tup1 shares structural and functional properties with Groucho, and is considered its homolog [2]. Tetrameric Tup1 forms a complex with Ssn6 and a variety of DNA-binding cofactors to modulate the transcription of hundreds of *S. cerevisiae* genes [6], [7]. The Tup1-Ssn6 complex is required for the repression of genes that are activated in response to alterations in growth conditions and cellular stresses. The Tup1-Ssn6 complex is targeted to promoters by DNA binding cofactors that are specific to the class of genes being repressed. For example, Tup1 is recruited to many glucose-repressed genes by Mig1 [8], to starch-degrading genes by Nrg1 [9], to osmotic-stress inducible genes by Sko1 [10], to hypoxia-repressed genes by Rox1 [11], to DNA-damage inducible genes by Rfx1 [12], to iron utilization genes by Aft1 [13], and to a peptide uptake gene by Cup9 [14]. In addition, Tup1 has been shown to physically interact with Sut1 [15], a regulator of sterol uptake and hypoxic gene expression [16], and participate in Tup1-dependent inhibition of transcription factor binding [17]. The Tup1-Ssn6 complex also plays a critical role in regulating cell-type-specific functions in yeast [18]. Specifically, in MATα haploid and MATa/MATα diploid cells Tup1 is recruited to and represses a-specific genes via the α2-Mcm1 heterodimer [19], [20], [21] and in diploid cells the a1-α2 heterodimer recruits Tup1 to repress haploid-specific genes [21], [22], [23].

The proteins that bring Tup1-Ssn6 to DNA vary in both their DNA-binding and protein-protein interaction domains. Tup1-Ssn6 recruitment and corresponding complex formation occurs by relatively weak protein-protein interactions with either Tup1 or Ssn6 [18]. Historically, recruiting cofactors have been identified by two criteria: their capacity to mediate locus-specific, Tup1-dependent repression and an ability to physically interact with Tup1 or Ssn6 [8], [9], [10], [11], [12], [13], [14], [19], [24]. Such locus-specific studies have characterized only a small subset of the more than 150 genes regulated by Tup1 [6].

To provide a more comprehensive model for genome-wide Tup1 recruitment, we used ChIP-chip assays to identify the genome-wide distributions of Tup1 and Ssn6. We then compared the binding pattern to published ChIP-chip data and found that the majority of loci bound by Tup1 were not co-occupied by a known Tup1 recruiter. In addition, individual deletions for seven known Tup1 recruiters did not significantly alter the Tup1 binding profile. These observations suggest that novel Tup1-Ssn6 recruiting proteins remain to be discovered and that Tup1 recruitment usually depends on multiple cofactors. To identify unknown Tup1 cofactors, we utilized an unbiased approach where we compared the genomic distribution of Tup1-Ssn6 to the distribution of more than 200 transcription factors. Using this method we identified several novel candidate cofactors along with the majority of the known Tup1-Ssn6 cofactors. Subsequently, we experimentally validated that the candidate cofactors Phd1, Cin5, Yap6, and Skn7 by showing that they physically interact with Tup1 and/or Ssn6. The newly identified cofactors are involved in regulating processes in which Tup1 has been previously implicated, including pseudohyphal growth (Phd1)[25], salt tolerance (Cin5, Yap6)[26], [27], and oxidative stress (Cin5, Skn7)[28], [29]. Accounting for these new recruiting proteins, we built a model that explains recruitment of Tup1 to the majority of its binding sites. Our approach, findings, and model for Tup1 recruitment both improve the understanding of Tup1 localization and regulation and provide a foundation for understanding the localization of eukaryotic repressor complexes.

## Results

### Tup1 binds to many sites in the absence of a known recruiter

**Figure 1:**
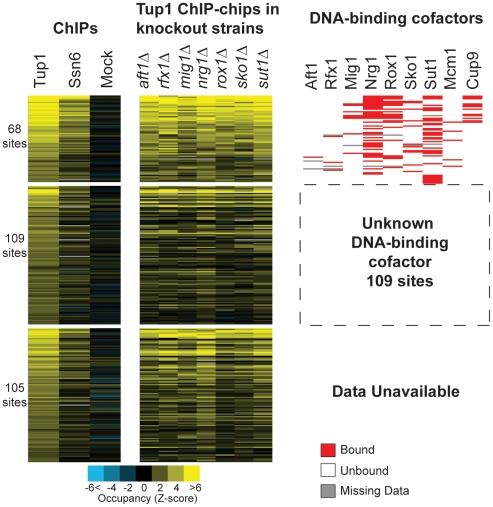
Only a fraction of sites bound by Tup1 in rich media are co-occupied by known cofactors. 282 sites bound by Tup1 during exponential growth in rich media were identified by ChIP-chip using a TAP-tagged protein and whole-genome tiled microarrays. For each target, the Z-score for the highest array element within the peak, as identified by ChIPOTle [55] is shown. Mock experiments performed in a wild-type strain lacking a TAP-tagged protein are also shown. The 282 Tup1 bound sites were sorted by p-value and split into three groups: 68 sites that are co-occupied by a known Tup1 recruiting protein (top), 109 sites not co-occupied by a known recruiting protein (middle), and 105 sites with no available cofactor binding data. Tup1 binding sites without cofactor data were located in regions of the genome that were not present on the microarrays used in the cofactor binding study [30]. Tup1 binding was also measured by ChIP-chip for strains carrying deletions of the known Tup1 recruiting cofactors Aft1, Rfx1, Mig1, Nrg1, Rox1, Sko1, and Sut1. DNA-binding cofactors are shown on the right, with occupancy as determined by ChIP-chip indicated in red (P<0.001 [30]). The following number of biological replicates were performed: Tup1, 21; Ssn6, 2; Mock, 4; recruiter deletion strains, 3–4.

To better understand how Tup1-Ssn6 is directed to its targets on a genome-wide scale, we first determined the genomic binding pattern of Tup1 and Ssn6 in yeast by ChIP-chip during growth in rich media (**Figure 1**). Targets were identified by hybridization to a PCR-based whole-genome DNA microarray covering all coding and intergenic regions at approximately 800-bp resolution. Tup1 was bound to 282 total targets (187 intergenic targets) with high confidence (FDR <0.001) as determined by 21 independent biological replicates (**Dataset S1**). For each peak of Tup1 binding, the highest-scored array element was used for all further analysis. We also determined the binding pattern of Ssn6 using two biological replicates. As expected, most Ssn6 targets (73%) were also bound by Tup1, and the Tup1 binding profile was highly correlated to Ssn6 occupancy (*R* ^2^ = 0.48). Also as expected, the majority of genes downstream of Tup1 binding events were derepressed in a *tup1Δ* strain (**Figure S1**) and included known targets such as the hexose transporters (GO term enrichment p = 7.05×10^−8^). The ChIP-chip results provided a high-confidence set of 282 Tup1 binding sites to use in down-stream analysis of Tup1 targeting.

To identify which cofactors occupied each of the 282 Tup1 target sites, we used published ChIP-chip data for the known Tup1 DNA-binding cofactors Aft1, Rfx1, Mig1, Nrg1, Rox1, Mcm1, Sko1, Cup9, and Sut1 [30]. The Harbison et al. (2004) experiments were performed on DNA microarrays that contained only the intergenic regions of the yeast genome; therefore, DNA-binding cofactor data was available for only 177 of the 282 identified Tup1 targets. Additionally, two well-characterized Tup1 recruiters (a1-α2 and α2-Mcm1) that regulate cell-type specific genes were not considered in this analysis because they are non-functional in the majority of the strains used for our study, and are non-functional in the published ChIP-chip studies. Surprisingly, 109 of the 177 (62%) Tup1 targets for which cofactor data is available were not co-occupied by a known Tup1 recruiting cofactor (binding defined at a p-value <0.001, **Figure 1**). Even when using a lenient cutoff of (P<0.01), no cofactor was bound at 42% of these Tup1 targets.

### Tup1 binding is not reduced by the deletion of individual Tup1 recruiters

**Figure 2:**
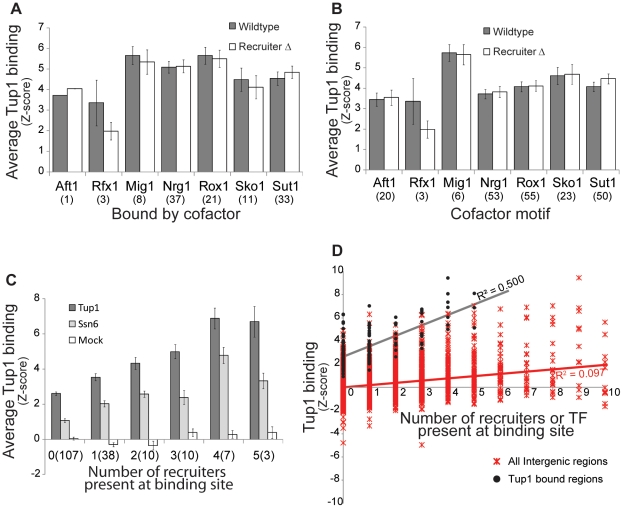
Deletion of individual Tup1 recruiters has little effect on Tup1 binding, and Tup1 binding is correlated with the presence of multiple recruiters. (A and B) The average enrichment (Z-score) in wild-type and recruiter deletion strains is plotted for (A) Tup1 targets bound (*P<*0.001) by a given transcription factor [30], or (B) Tup1 targets containing a binding site (P<0.005) for a given transcription factor [31]. To control for differences in IP efficiency between experiments Tup1 binding values were standardized to wild-type Tup1 binding by scaling Tup1 binding in each deletion strain so that the average Tup1 occupancy across all bound regions is the same for all experiments. The number of Tup1 targets in each group is indicated in parentheses and error bars represent standard error. (C) Tup1 targets were binned based on the number of recruiters bound to the target (P<0.001; [30]). Average enrichment (Z-score) for Tup1, Ssn6, or mock ChIPs for each group was calculated. The number of targets in each group is indicated in parentheses and error bars represent standard error. (D) Tup1 occupancy is plotted for Tup1 bound regulatory regions as a function of the number of bound recruiter proteins (Black) and for all regulatory regions, including the Tup1 bound regions, (Red) as a function of the number transcription factors bound at the regulatory region. The linear regression line and coefficient for both datasets is shown.

To further explore the mechanism of Tup1 recruitment to specific targets, we examined Tup1 binding in seven strains, each carrying a deletion of a different known recruiter of the Tup1-Ssn6 complex. Then, for each Tup1 target, we compared the wild-type ChIP values to the values obtained in each of the seven deletion strains (**Figure 1**). On a qualitative level, deletion of each of the recruiters had little effect on Tup1 binding. To quantify our results, we split the Tup1 targets into groups. First, all Tup1 targets that were bound by each of the cofactors were identified [30] **(** **Figure 2A** **)**. Next, all of the DNA sequences near sites of Tup1 binding were screened for the presence of DNA sequence motifs that corresponded to the specificities of each of the recruiters [7], [31] (**Figure 2B**). For each of these categorizations a given target may be in more than one group, for example a Tup1 target bound by both Sut1 and Mig1. We then compared Tup1 binding in wild-type cells and the appropriate deletion strain for each group. The results were clear: there was no significant change in Tup1 binding at sites that contained the deleted recruiter’s motif, nor was there any change in Tup1 binding at sites normally co-bound by the deleted recruiter (**Figure 2A and B**). Thus, for the majority of Tup1 targets, deletion of a single known recruiter did not eliminate or significantly alter Tup1 binding. This suggests that multiple redundant recruiters direct Tup1 to each of its targets, or possibly that when a single recruiter was deleted another operates in its place.

### Evidence that Tup1 is recruited by multiple cofactors at many of it sites

To test the hypothesis that multiple recruiters direct Tup1 to its targets, we once again divided Tup1 targets into groups, this time based on the number of different recruiters bound to each target. We then calculated the average Tup1 binding score for each group of targets. We observed a positive relationship between the magnitude of either Tup1 or Ssn6 binding signal and the number of recruiters bound to the target, which was not seen for mock ChIP experiments (**Figure 2C**). To further explore this relationship, we preformed a linear regression for Tup1 binding by the number of recruiter proteins bound and discovered a highly significant regression of *R* ^2^ = 0.500 (P<1×10^−10^) (**Figure 2D** **)**. To ensure that this correlation was specific to recruiters of Tup1, we calculated the regression coefficient between Tup1 occupancy at all intergenic regions to the total number of proteins bound using all the ChIP data, and found the relationship to be greatly reduced (*R* ^2^ = 0.097). The strong association between the number of recruiting proteins present and Tup1 binding signal suggests that Tup1 is simultaneously recruited by multiple cofactors to its targets.

### Identification of new candidate Tup1 recruiters

**Figure 3:**
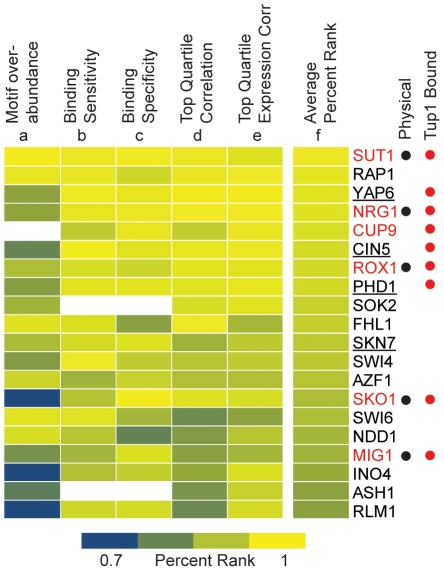
Prediction of novel Tup1 recruiters. Tup1 binding data was compared to ChIP-chip data from 204 transcription factors [30], [31] and *tup1Δ* expression data [7] using five tests (see text and Material and Methods for details). The percentile rank for each test and average percentile rank across all five tests is shown for the top 20 candidate transcription factors. Known Tup1 recruiting proteins are indicated by red text. Transcription factors previously shown to physically interact with Tup1 or Ssn6 are indicated with a black dot [57] and the transcription factors whose own promoters are bound by Tup1 are indicated by a red dot. Predicted Tup1 recruiters that were studied further are indicated with underlined text.

Despite evidence for multiple recruiters, over 40% of Tup1 genomic targets were not bound by a known recruiter, even when using a relatively liberal cutoff (P<0.01). Thus, either Tup1 binds directly to some of its targets without assistance, possibly through association with histone tails [32], or additional transcription factors are capable of recruiting the Tup1-Ssn6 complex, or both may be true. To address this question, we utilized ChIP-chip data for 204 transcription factors [30], [31] and expression data from at *TUP1* deletion mutant [7] to identify potential novel Tup1 recruiters. We performed five predictive tests detailed in *Materials and Methods* (**Figure 3**). Briefly, we (a) Calculated the over-representation of each transcription factor’s DNA-binding motif among Tup1 targets. (b) Calculated the percentage of Tup1 targets that were bound by each transcription factor. (c) Calculated the percentage of each transcription factor’s targets that are bound by Tup1. (d) Calculated the correlation between the top quartile of Tup1 binding and the top quartile of binding for each transcription factor. (e) Calculated the correlation between the top quartile of expression changes in a *tup1Δ* and the top quartile of binding for each transcription factor. We then assigned each of the 204 transcription factors a percentile score for each test, and calculated the average percentile (f) across all five tests for each transcription factor.

There are three readily apparent biological explanations for why a transcription factor might score highly in our prediction tests. First, Tup1 may modulate the binding of the transcription factor to some of the transcription factor’s targets. Second, Tup1 may independently co-regulate some of the transcription factor’s targets. Third, the transcription factor may recruit Tup1 to a subset of its targets or be a member of the Tup1-Ssn6 complex. We are interested in this third class.

Multiple lines of evidence suggest that some of the predicted recruiters do in fact function to direct Tup1 to its target genes. First, six of the eight (Sut1, Nrg1, Rox1, Sko1, Mig1, and Cup9) previously characterized Tup1 recruiters for which we have data rank in the top twenty of the 204 tested transcription factors. Second, we have observed that Tup1 itself binds to the regulatory regions upstream of nine of the top 20 genes, including all six of the known recruiters on the list (Sut1, Nrg1, Rox1, Sko1, Mig1, and Cup9) (**Figure 3** red dots). This suggests that Tup1 may regulate its own activity by modulating the expression of its recruiters in a feed-forward network.

### Four of the newly predicted Tup1 recruiters physically interact with Tup1-Ssn6

We further studied four of the proteins that scored highly in our predictive tests (Cin5, Phd1, Yap6, and Skn7; **Figure 3**, underlines) to determine if they recruit Tup1. While Cin5, Phd1, Yap6, and Skn7 are generally characterized as activators of transcription rather than repressors, they all regulate genes within Tup1 characterized pathways. For example, Cin5 has previously been implicated in Tup1-mediated repression through network analysis [33], and Tup1 and Yap6 were proposed to regulate a common set of genes based on analysis of expression in a *tup1* mutant. [34].

**Figure 4:**
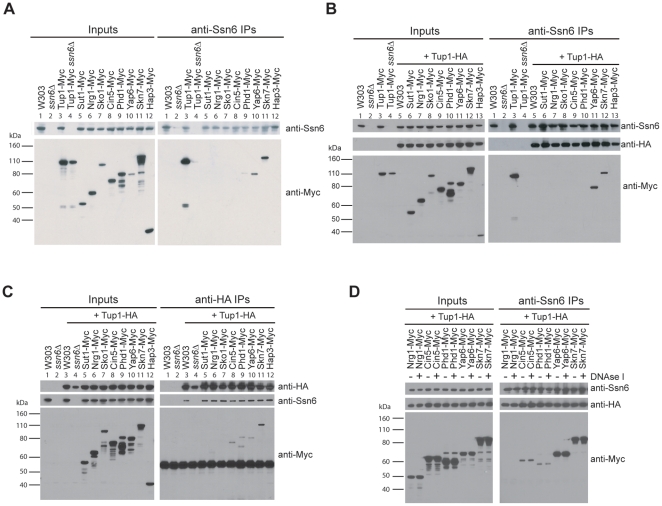
Candidate recruiters physically interact with Tup1-Ssn6. Strains carrying Myc-tagged candidate recruiters (Cin5, Phd1, Yap6, or Skn7), previously characterized recruiters (Sut1, Nrg1, or Sko1), or a protein that was not predicted to interact with Tup1 (Hap3) were immunoprecipitated with anti-Ssn6 antibodies (A and B), or with anti-HA antibodies (to immunoprecipitate HA-tagged Tup1) (C). Inputs (left) and immunoprecipitated material (right) were immunoblotted with anti-Ssn6 antibody, anti-HA antibody (to detect Tup1), or anti-MYC (to detect recruiter proteins). (D) The interactions between Tup1-Ssn6 and predicted recruiters are not mediated by DNA. Strains carrying a Myc-tagged characterized recruiter (Nrg1) or predicted recruiters (Cin5, Phd1, Yap6, or Skn7) were immunoprecipitated with anti-Ssn6 antibodies in the presence (+) or absence (−) of DNAse I. Inputs (left) and immunoprecipitated material (right) were immunoblotted with anti-Ssn6 antibody (top), anti-HA antibody (middle) or anti-MYC (bottom).

If our candidates are true Tup1 cofactors, we expect that they will physically interact with Tup1-Ssn6. To test this, we performed a series of *in vivo* co-immunoprecipitation (co-IP) experiments in strains harboring Myc-tagged versions of the potential Tup1 recruiters. In the first experiment, we immunoprecipitated with anti-Ssn6 antibodies and then immunoblotted with anti-Myc to determine whether Cin5, Phd1, Yap6, and Skn7 were associated with Ssn6. As a positive control, we confirmed the ability of Ssn6 to immunoprecipitate Tup1-MYC in an Ssn6-dependent manner (**Figure 4A** compare anti-MYC IP blot lanes 3 and 4). Skn7 and Yap6 exhibited a strong interaction with Ssn6, while Phd1 exhibited a weaker interaction (**Figure 4A** lanes 9, 10, and 11). Hap3, a protein that had low scores for all of our predictive tests, showed no interaction with Ssn6. However, the known Tup1-cofactors Sut1, Nrg1, and Sko1 and the newly predicted recruiter Cin5 were also not detected in this co-IP experiment.

To confirm the strong interaction of Skn7 and Yap6 with Tup1-Ssn6, we repeated our co-IP experiments. For these experiments we added a HA-tag to Tup1 in all of the strains harboring a MYC-tagged recruiter protein to confirm that the Tup1-Ssn6 complex was intact throughout our IP experiments (**Figure 4B**). This second set of co-IPs showed that Tup1 and Ssn6 were always pulled down together, and confirmed that Skn7 and Yap6 interact with Tup1-Ssn6. With a longer exposure of the Myc Western blot, interactions are detectable for our three positive controls, Sut1, Nrg1, and Sko1, in addition to our two other top candidates, Cin5 and Phd1, while Hap3 continues to show no interaction with Ssn6 (**Figure S2**). The signal produced by the Cin5 and Phd1 is comparable to that of the known recruiters.

In a third experiment, we immunoprecipitated Tup1-HA instead of Ssn6 and identified proteins that were immunoprecipitated along with Tup1. Again, we confirmed that the Tup1-Ssn6 complex was intact throughout our experiments by showing that Ssn6 is consistently co-immunoprecipitated with Tup1-HA. Tup1 exhibited a strong interaction with Skn7 and weaker interactions with Yap6, Cin5, Phd1 and the previously known recruiter Nrg1 (**Figure 4C**). In this experiment, we failed to identify a co-IP interaction between Tup1-HA and Sko1. Detection of Sut1 was not possible in this experiment because the Sut1 band was obscured by the IgG band.

### The interactions of Cin5, Phd1, Yap6, and Skn7 with Tup1-Ssn6 are not mediated by DNA

The apparent interactions between the predicted recruiters and Tup1-Ssn6 could result from the proteins occupying the same regulatory region and being bridged by DNA rather than by direct protein-protein interaction. To address this issue, we repeated the co-IP experiments with extract treated with DNAse I for 30 minutes prior to the immunoprecipitation. While gel electrophoresis and PCR analysis indicate that the DNA was digested to near completion (**Figure S3**), treatment with DNAse I did not prevent the ability of Ssn6 to pull down Yap6, Skn7, Phd1, or Cin5 (**Figure 4D**). Thus, the interaction is not likely to be mediated by DNA, but instead is a protein-protein interaction with the Tup1-Ssn6 complex. Taken together, the co-IP experiments show that the newly predicted Tup1-Ssn6 recruiters Skn7, Yap6, Phd1, and Cin5 all physically interact with Tup1.

### Tup1-Ssn6 functions as a repressor at sites bound by Yap6, Skn7, Phd1, and Cin5

**Figure 5:**
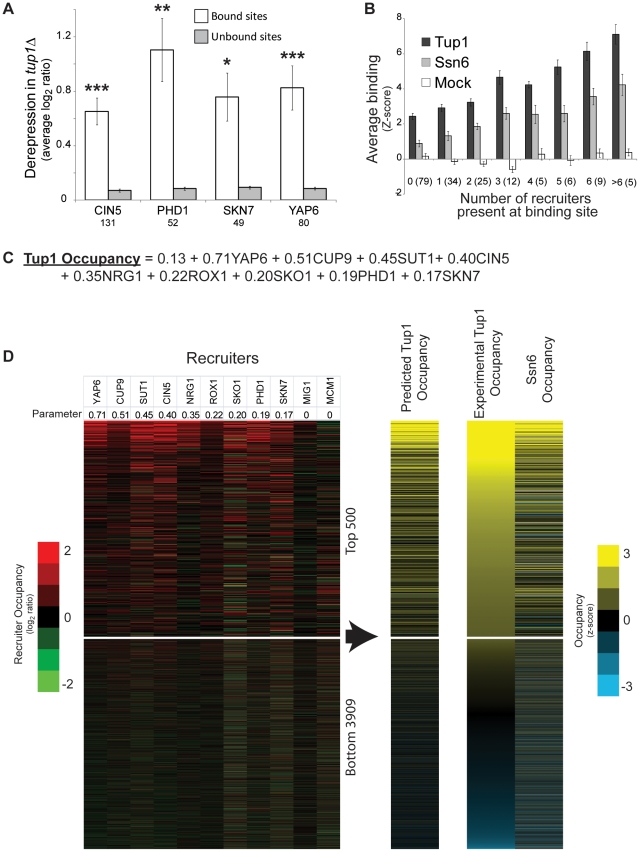
New Tup1 recruiters improve the model for Tup1 recruitment. (A) The average downstream gene expression for the targets (P<0.001) and non-targets (P>0.05) of the four new recruiters in *tup1Δ* strain is plotted [7]. The number of sites is listed and error bars represent standard error. The significance for the difference between the bound (P<0.001) and unbound sites (P>0.05) was determined by t-Test; * P<0.001, ** P<1×10^−4^, *** P<1×10^−5^. (B) Tup1 targets were separated based on the number of recruiters bound (P<0.001; [30]), including the four new candidate recruiters (Cin5, Phd1, Yap6, and Skn7). The average enrichment (Z-score) for Tup1, Ssn6, or Mock experiment for each group is shown. The number of targets in each group is shown in parenthesis and error bars represent standard error. (C) Regression model including new candidate recruiters. This model describes how recruiter occupancies, as measured by ChIP-chip, can be used to predict Tup1 occupancy at a genomic region. (D) The log_2_ ratio for each Tup1 recruiting protein is shown on the left, with the parameter estimate for the regression model on top. Tup1 binding predicted by the model is shown with the experimental Tup1 and Ssn6 occupancy. All data is sorted by the experimental Tup1 occupancy.

To determine if the Tup1-Ssn6 complex is functioning as a repressor at sites bound by Yap6, Skn7, Phd1, and Cin5, we calculated change in gene expression at each bound group (P<0.001) [30] compared to unbound sites (P>0.05) in a *tup1Δ* strain [7]. In a *tup1Δ* strain, genes downstream of sites bound by any of the four proteins were strongly derepressed (**Figure 5A**), indicating that Tup1 is functioning as a repressor at these sites and suggests that Yap6, Skn7, Phd1, and Cin5 are likely co-repressors with Tup1-Ssn6. This observation is consistent with a previous analysis that indicated that the targets of Yap6, Skn7, Phd1, and Sok2 (an untested candidate Tup1 recruiter) are all significantly derepressed in strains deleted for Tup1 or Ssn6 [33].

### Yap6, Cin5, Skn7, and Phd1 improve models of Tup1-Ssn6 targeting

To further validate the role of these newly predicted Tup1 recruiters in Tup1-Ssn6 targeting, we performed our analyses from **Figure 2C and 2D** incorporating these new proteins. Including Yap6, Cin5, Skn7, and Phd1 as recruiter proteins strengthens the positive relationship between the number of recruiters bound to a given locus and the level of Tup1 and Ssn6 enrichment observed (**Figure 5B**) (**Dataset S2)**. Additionally, the regression between the number of binding recruiters and Tup1 occupancy improves from an *R* ^2^ of 0.50 to 0.58 when these four proteins are included (same analysis as **Figure 2D**). This strongly suggests that the new discovered recruiters participate with the previously identified cofactors in recruiting the Tup1-Ssn6 complex.

It is likely that all Tup1-Ssn6 cofactors recruit the complex with different efficiencies, and it is further likely that the efficiency of recruitment varies from locus to locus depending on the DNA sequence and other factors. To more accurately model Tup1-Ssn6 complex recruitment in rich media we generated a regression model for Tup1 occupancy using the experimentally measured occupancy of the recruiters at each regulatory region. This model allows recruiters to have differing recruitment strengths, and also incorporates how strongly each recruiter associates with a regulatory region.

Regression analysis examines the relationship between one variable and another set of variables. The relationship is expressed as an equation that predicts a response variable (Tup1 occupancy) from a function of regressor variables (Tup1-Ssn6 recruiting proteins) and parameters. The parameters are adjusted so that a measure of fit is optimized. There is a single parameter for each recruiter protein in the model that defines the weight given to a recruiter protein in the regression model. A value close to zero has low weight while a large positive or negative parameter value is weighted highly for its ability to predict Tup1 occupancy.

We first tested each recruiter independently to determine if each single recruiter was significantly positively correlated to Tup1 occupancy. At this step, we removed from the model Rfx1, which did not have a significant relationship with Tup1 binding, and Aft1, which was negatively correlated (See Discussion). Using only the previously known Tup1-recruiting proteins (Cup9, Mcm1, Mig1, Nrg1, Rox1, Sko1, and Sut1) we generated a regression model on half of the genome, and then validated the model on the remaining half. The model reports that the presence of Cup9, Rox1, Sut1, Nrg1, and Sko1 all contribute to the association of Tup1 with its genomic targets. The occupancy of Mig1 and Mcm1 do not contribute significant additional information to determining the binding pattern of Tup1 (**Table 1**). Therefore, Mig1 and Mcm1 can be removed from the model without sacrificing its predictive power (See Discussion).

**Table 1:** Regression results for both the initial and final model.

	Intercept	SUT1	NRG1	ROX1	SKO1	CUP9	MIG1	MCM1	YAP6	CIN5	PHD1	SKN7	*R* ^2^
**Initial Model**	0.105	0.656	0.588	0.644	0.263	0.772	0.086	0.006					**0.33**
**p-value**	<0.0001	<0.0001	<0.0001	<0.0001	<0.0001	<0.0001	0.0864	0.8553					
**Final model**	0.129	0.454	0.348	0.215	0.198	0.513	0.053	−0.029	0.706	0.395	0.190	0.170	**0.43**
**p-value**	<0.0001	<0.0001	<0.0001	0.0002	<0.0001	<0.0001	0.2618	0.344	<0.0001	<0.0001	<0.0001	<0.0001	

We next determined if a new regression model that includes the known Tup1 recruiters and our top candidates, Yap6, Phd1, Cin5, and Skn7, was better able to predict Tup1 experimental occupancy than the model containing the known recruiters alone (**Figure 5C**). We found that the addition of each protein into the model results in significant improvement (**Table 1**). By incorporating all of the newly identified recruiters, the prediction of Tup1 occupancy at Tup1 bound sites improved from an *R* ^2^ of 0.577 to 0.648 (p-value  = 2.3×10^−17^) (**Dataset S3**). Overall, this means that our model can explain 65% of Tup1 binding variance at Tup1 bound sites and 43% of variance in Tup1 binding for all yeast intergenic regions (**Figure 5D**). This is remarkable because a significant proportion of the binding variance is likely due to technical noise in the ChIP-chip experiments, which were performed using low-resolution spotted-DNA microarrays. This noise would be present in both the predictor and the response variables. By comparison, our ChIP-chip of Ssn6, which is thought to be in tight association with Tup1 at all times on genomic DNA and theoretically would predict Tup1 binding perfectly, does only slightly better than our model, explaining 48% of the Tup1-binding variance across the genome. This suggests that our model is approaching the maximal possible predictive value achievable with these datasets. Finally, the addition of Cin5, Phd1, Skn7, and Yap6 increased the proportion of Tup1 binding sites also occupied by a recruiting protein from between 38% and 58% (**Figure 1**) to between 55% and 73% depending on the cofactor binding p-value used to define “occupied” (<0.001 or 0.01 respectively).

## Discussion

We have demonstrated that Tup1 is bound to 282 loci across the yeast genome and shown that deletions of individual recruiters did not significantly change the Tup1 binding pattern. We also identified a correlation between Tup1 occupancy and the number of recruiting proteins bound at a given regulatory region. This is consistent with a study showing that the Tup1 recruiters Rox1 and Rfx1 together with a third protein Mot3 act synergistically to promote Tup1 mediated repression [35]. Our results indicate that this cooperation in directing Tup1 binding is likely more wide spread phenomena.

Prior to our study, no known Tup1 recruiter was present at 62% of Tup1-bound sites. We utilized computational approaches to identify new candidate recruiters. These approaches predicted that Yap6, Cin5, Phd1, and Skn7 may act as Tup1 recruiters, and we experimentally verified that these proteins physically interact with Tup1-Ssn6. Additionally, genes bound by Yap6, Cin5, Phd1, and Skn7 were shown to be strongly de-repressed upon knockout of Tup1.

### The newly identified Tup1 recruiters are functionally linked to stress response

Each of the new Tup1-Ssn6 cofactors has been previously characterized to function in a variety of stress and growth responses processes that Tup1 is known to regulate [36]. Cin5 and Yap6 are closely related AP-1 factors that belong to a fungus-specific family of bZIP proteins [26]. Their binding patterns are highly correlated, targeting 73% to 86% of the same sites, depending on the growth conditions [37]. Cin5 and Yap6 are likely involved in yeast stress response, because overexpression of Cin5 or Yap6 increases tolerance to sodium, lithium, and cisplatin [27], [38], and overexpression of Cin5 confers resistance to quinidine, mefloquine, and chloroquine [39]. While these proteins are often considered transcriptional activators, there is evidence that many of them also have repressive functions. Yap6 binding has been shown to occur at activated and repressed genes in response to exposure to increased salt [37]. Skn7 is required for the oxidative stress response in yeast [40], [41] and appears to function as a transcriptional activator with Yap1 to activate oxidative stress response genes [42]. However, Skn7 may also have repressor activity, since increased binding of Skn7 is associated with repression during salt response [37]. Phd1 has been identified as key hub protein for the regulation of pseudohyphal growth [43], and over expression of Phd1 causes the activation Flo11 and the induction of pseudohyphal growth [25].

### The new recruiters allow us to explain up to 73% of Tup1’s targeting

Inclusion of new recruiters increases the correlation between the number of distinct recruiters bound and Tup1 occupancy for all yeast intergenic regions (**Figure 5B**). Furthermore, the new recruiters increase the number of Tup1 targets also bound by a recruiter protein from 58% to 73%, helping to fill gaps in our understanding of Tup1-targeting. Importantly, incorporating the four new recruiters with the previous characterized recruiters into a regression model for Tup1 binding explains 65% of Tup1 occupancy at Tup1 bound sites, and strengthens the case for simultaneous recruitment of the Tup1 complex by multiple DNA binding cofactors. While inclusion of the newly identified Tup1 recruiters does not explain all Tup1 binding, our results do increase the number of Tup1 binding events explained by the presence of a recruiter by 15% or more (depending on cutoff used to define binding events) and explain a significantly greater proportion of the Tup1 occupancy variance. Additionally, we were able to experimentally validate 7 of the top 10 computationally identified candidate cofactors, suggesting that some other highly-ranked proteins on our list may also function in association with Tup1-Ssn6. Rap1 and Sok2, which we were not able to test, are good candidates. At individual sites, Tup1-Ssn6 is recruited simultaneously by multiple proteins and is likely forming a multi-meric complex containing Tup1, Ssn6, multiple recruiting proteins, and chromatin remodeling factors.

### Strengths and limitations of the Tup1 recruitment model

Our model can only be as accurate as the datasets used for predictions and for determining Tup1 occupancy. Any noise or biases in the datasets will generate noise in our model. In addition, our model did not incorporate the possibility that Tup1 binds directly to deacetylated histone tails [44]. It is likely that use of higher resolution binding data, inclusion of a parameter to allow Tup1 to bind directly to histone tails, and the discovery of additional Tup1 recruiting proteins will improve our model when incorporated. Nonetheless, our regression model including the new cofactors, describes a significant percentage (43%) of Tup1 binding variance across the entire genome.

To develop our model we initially tested each recruiter to determine if their binding was significantly positively correlated with Tup1 occupancy. Rfx1 was not significantly correlated and Aft1 was negatively correlated with Tup1 occupancy. The significant negative correlation of Aft1 suggests Aft1 negatively predicts Tup1 binding in rich media, which is inconsistent with the function of a Tup1 recruiter. However, Aft1 does not recruit Tup1 during growth in rich media because it is restricted to the cytoplasm in this growth condition [45]; therefore it is not surprising that Aft1 is not correlated with Tup1 localization under the conditions tested in this experiment. On the other hand, Rfx1 recruits Tup1-Ssn6 to DNA damage inducible genes to repress transcription in the absence of DNA damage signals [35]. Therefore, Rfx1 should be actively recruiting Tup1-Ssn6 in the conditions tested for these experiments. The lack of a significant correlation between Rfx1 binding and Tup1 is likely do to noise in the datasets or the limited number of genomic loci for which Rfx1 recruits Tup1.

Our multi-recruiter model of Tup1 binding was generated by regression analysis. Regression analysis creates an equation describing how all the recruiters in combination predict Tup1 occupancy. In this analysis, if a recruiter’s targets are completely contained within another recruiter’s targets, then its parameter or weight in the model will be reduced. Our final model showed that the presence of Yap6, Cup9 Sut1, Cin5, Nrg1, Rox1, Sko1, Phd1, and Skn7 all contributed to the association of Tup1 with its genomic targets, while the occupancy of Mig1 and Mcm1 do not contribute additional information. It is not surprising that Mcm1 binding from a MATa strain of yeast does not significantly help predict the location of Tup1, because α2-Mcm1 only recruits Tup1-Ssn6 in MATα yeast [21]. On the other hand, Mig1 should be functioning to recruit Tup1 in the strains tested in our study, but was removed from the regression model only after the inclusion of other cofactors. This could be caused if, for example, Mig1 binds and recruits Tup1 with another cofactor already in the model, or the published Mig1 ChIP-chip data is of lower quality than the other ChIP-chip datasets.

### Integrating multiple environmental inputs at gene promoters

How does the Tup1-Ssn6 repressor receive signals from multiple signaling pathways, integrate those signals, and regulate gene expression accordingly? The activities of Tup1 recruiting proteins are modulated by signaling pathways that are activated by an overlapping set of environmental conditions and cellular stresses. These pathways include the hexokinase 2 signaling pathway, Snf1 signaling pathway, the Hog protein kinase pathway, and the Mec1 kinase cascade [12], [46], [47], [48]. Tup1 and its recruiting proteins integrate the signals from these pathways using a number of mechanisms. For example, the Snf1 signaling pathway regulates Mig1 localization [49]. In the presence of glucose, Mig1 recruits Tup1 to its targets to repress their transcription, while in low glucose Snf1 phosphorylates Mig1, resulting in Mig1 export from the nucleus and expression of its target genes. In contrast, the Hog kinase pathway regulates Sko1 by converting it from a repressor to an activator [48]. In normal growth conditions, Sko1 recruits Tup1 to its targets and represses their transcription, while osmotic stress causes Hog1 to phosphorylate Sko1, resulting in the recruitment of SAGA and SWI/SNF and the activation of its targets. Interestingly, full activation of these genes requires the presence of Tup1 [48], [50]. Consistent with this observation, we have shown previously that Tup1 remains bound to many targets even when transcriptional repression is relieved [17].

Years of work from numerous labs studying Tup1 and its recruiters together with our results suggest that Tup1, Ssn6, and multiple recruiting proteins form a repressor complex that prevents the expression of hundreds of genes that are not required under normal laboratory growth conditions [6], [18], [51]. The relationship between Tup1-Ssn6 and its recruiters allows the cell to respond quickly and specifically to a given stress, without, for example, derepressing all gene targets of Tup1. It is likely that the function of the Tup1-Ssn6 complex at an individual site depends heavily on the context of the promoter and the suite of cofactors assembled.

## Materials and Methods

### Yeast strains

The genotypes and sources of the strains used in this study are listed in Table S1. SHy048 was generated by first mating SHy028 with BY4742, sporulating the resulting diploid and selecting a MATα colony containing the Tup1-TAP::HIS3 allele. All other epitope tagged and deletion strains were generated by one-step gene replacement as previously described [52].

### Chromatin Immunoprecipitations

Vegetative samples were grown in YPD (1% yeast extract, 2% peptone, 2% dextrose) to an OD_600_ of 0.6–0.8. ChIPs for TAP-tagged Tup1 were performed as before [17] with minor modifications. The function of Tup1 when tagged with TAP was confirmed by examining Tup1 repression of the FLO1 gene (data not shown). Briefly, 1% formaldehyde-fixed cells were resuspended in FA-Lysis buffer (50 mM Hepes-KOH, pH 7.5, 300 mM NaCl, 1 mM EDTA, 1.0% Triton-X, 0.1% Sodium deoxycholate, and 1 X protease inhibitors (Calbiochem)) and disrupted using a Mini-Beadbeater-8. The isolated chromatin was sheared to an average size of 0.8 kb and incubated overnight at 4°C with IgG sepharose beads to recover Tup1-TAP associated DNA. The beads were washed two times each with FA lysis buffer, FA Wash 2 (50 mM Hepes-KOH, pH 7.5, 500 mM NaCl, 1 mM EDTA, 1.0% Triton-X, 0.1% Sodium deoxycholate), FA Wash 3 (50 mM Hepes-KOH, pH 7.5, 250 mM LiCl, 1 mM EDTA, 1.0% Triton-X, 0.1% Sodium deoxycholate) and TE each supplemented with 1 X protease inhibitors. ChIP Elution Buffer (50 mM Tris-Cl pH 8.0, 10 mM EDTA, 1% SDS) was added to the washed beads and Tup1-TAP associated DNA fragments were eluted by incubation for 1 hour at 65°C. Eluted ChIP samples (and inputs) were incubated at 65°C overnight to reverse cross-links, excess protein was degraded using Proteinase K and DNA was isolated using Zymo columns according to the manufacturer’s instructions (Zymo Research).

### DNA amplification and labeling

ChIP and input DNA were amplified by ligation-mediated (LM) PCR as described previously [53]. Briefly, recovered ChIP and input DNA were blunted using T4 DNA Polymerase (NEB) and unidirectional linkers were ligated to the ends of the DNA fragments using T4 DNA Ligase (NEB). The long oligonucleotide from the unidirectional promoter was then used to amplify the DNA in two steps of PCR for a total of 33 cycles. In the second PCR step (20 cycles) amino-allyl dUTP (Sigma) was incorporated at a ratio of 3∶2 with dTTP. Reactive Cy3 or Cy5 (Amersham) was coupled to the amino-allyl of the resulting DNA fragments in the presence of sodium bicarbonate.

### Microarray hybridizations and image acquisition

ChIP samples were competitively hybridized with input DNA to low-resolution spotted-DNA yeast whole genome spotted microarrays containing coding and non-coding regions at approximately 800 bp resolution. The arrays were scanned with an Axon 4000 scanner, and data was extracted using GenePix 6.0 software. Only spots of high quality by visual inspection, with less than 10% saturated input pixels, with a background corrected sum of medians for both channels greater than 500 were used for the analysis.

### Array normalization and quality control

The DNA microarrays were normalized using block-by-block loess with the limma R package as part of Bioconductor [54]. Initially the complete dataset contained 56 arrays generated by 3 researchers in both dye-orientations. All of the arrays were accessed for quality and bad arrays were removed due to regional artifacts, inefficient labeling, or low correlation to other arrays in the experimental group. The final 41 arrays have been uploaded to the GEO database accession GSE26311, and supplemental files are available at http://buffalo.edu/mjbuck/Tup1-recruiters.html.

### Determination of binding sites

The median standardized value was determined across all biological replicates. The standardized log ratio was used as input for ChIPOTle [55] with the following parameters: Gaussian background distribution, step size 0.25 kb and window size 1 kb. Peaks with FDR cutoff <0.001, a Z-score >1 above mock control, and a mock Z-score <2 were retained for further analysis. Neighboring peaks within 1 kb were collapsed and the highest bound array element was represented in the cluster-grams and used for all further analysis.

### Prediction of novel recruiters

Starting with our Tup1 ChIP-chip data we utilized published ChIP-chip data for 204 transcription factors [30], [31] and *tup1Δ* expression data [7] to identify potential novel Tup1 recruiters. We performed five separate prediction tests: (a) Calculated the over-representation of each transcription factor’s DNA-binding motif among Tup1 targets compared to the rest of the genome. Motif over-representation was determined using the Relative OVER-abundance of cis-elements (ROVER) algorithm with a p-value cutoff of 0.001 [56] and previously reported position-weight-matrixes (PWM) [31]. (b) Calculated the percentage of Tup1 targets that were bound by each transcription factor (i.e. sensitivity). Sensitivity was calculated by dividing the number of binding events for a transcription factor (at a p-value <0.001; [30]) that fall within Tup1 targets by the total number of Tup1 targets. (c) Calculated the percentage of each transcription factor’s targets that are bound by Tup1 (i.e. specificity). Specificity was calculated by dividing the number of binding sites for a transcription factor (at a p-value <0.001) [30] that fell within Tup1 targets by the total number of targets for the transcription factor (at a p-value <0.001) [30]. (d) Calculated the correlation between the top quartile of Tup1 binding and the top quartile of binding for each transcription factor. This approach allowed us to examine the relationship between Tup1 and possible recruiters, without rigidly selecting bound Tup1 targets, and it determined which possible recruiters’ binding pattern changes in a similar manner as Tup1 binding pattern. If a protein is in fact a Tup1 recruiter, its occupancy at any site in the genome should be positively correlated to Tup1 occupancy at that site. Although this approach assumes inaccurately independence between recruiters, it still identifies the strongest correlated components. (e) Calculated the correlation between the top quartile of expression changes in a *tup1Δ* and the top quartile of binding for each transcription factor. The last approach is similar to the fourth but instead of using *in vivo* Tup1 binding data we used the change in expression for downstream genes in a *tup1Δ* [7]. Each transcription factor was assigned a percent rank for each test and (f) the average percent rank across all five tests was calculated. The average was calculated based only on those tests for which a transcription factor could be included. All 204 transcription factors were sorted based on the average percent rank.

### Regression analysis

Regression analysis examines the relationship between one variable (Tup1 occupancy) and another set of variables (Tup1-Ssn6 recruiting proteins). The data for each recruiter was the log_2_ ratios from ChIP-chip experiments [30] and missing data was simulated by a Gaussian random function. The simulation was repeated 10 times, and was demonstrated to have a limited effect on parameter estimates. We first determine which previous characterized recruiting proteins (Aft1, Rfx1, Mcm1, Mig1, Nrg1, Rox1, Sko1, Cup9, and Sut1) independently, and positively, predicted Tup1 occupancy. Based on this initial analysis Aft1 and Rfx1 were removed from further consideration. The six remaining variables were then fit to a regression model using a random half of all yeast intergenic regions and validated by repeated random sub-sampling cross-validation. The validated model was then tested on the remaining half of the yeast intergenic regions to estimate its *R* ^2^. The yeast intergenic dataset was then randomly split 4 additional times and the regression model estimation was repeated. The final parameter estimates were determined as the average across all five regression models. The final regression model was determine as outlined above but included the additional Tup1-Ssn6 recruiting proteins (Cin5, Phd1, Skn7, and Yap6). Mcm1 and Mig1 were removed from the final model because they were both non-significant predictors (p>0.01). All regression analysis was performed using SAS v 9.1 (SAS Cary, NC).

### Co-Immunoprecipitations

Appropriate strains were inoculated and grown in YPD to an OD_600_ of 0.5–1.0 and for each strain, 25–30 OD_600_ units were collected at 4°C (all remain steps performed at 4°C). Pellets were washed with Co-IP buffer (50 mM Tris-Cl pH 7.4, 250 mM NaCl, 5 mM EDTA, 0.1% NP40; for DNAse I experiments EDTA was omitted) plus protease inhibitors (1x protease inhibitor cocktail, 1.5 mM DTT, 1 mM PMSF, 1 mM Benzamidine), transferred to ependorf tubes, and lysed with glass beads. The lysates were removed from the glass beads and cleared by spinning at 15,000 RPM for five minutes. For the DNAse I experiments, DNAse I was added to a final concentration of 100 U/µl, samples were incubated for one hour at 4°C, and EDTA was added to a final concentration of 10 mM to stop reactions. For inputs, 25 µl of cleared lysates was added to 25 µl of 2X sample buffer (125 mM Tris-Cl pH 6.8, 20% glycerol, 4% SDS, 10% beta-mercaptoethanol). For IPs, antibody (either 1 µl of anti-Ssn6 antibody or 5 µl of anti-HA antibody) was added to 500 µl of cleared lysates and incubated for three hours at 4°C. Antibody-protein complexes were recovered by incubation with Protein G Sepharose beads in Co-IP Buffer, beads were washed two times with Co-IP Buffer and once with high-salt Co-IP Buffer (50 mM Tris pH 7.4, 500 mM NaCl, 5 mM EDTA, 0.1% NP40). Following the final wash, all buffer was removed from the beads and 20 µl of 2X sample buffer was added to the beads. For detection of proteins by Western blot, 5 µl of each input was loaded for each blot (approximately 0.5% of material used in IP). For anti-Ssn6 co-IP blots, 10 µl was loaded for anti-Myc blots (approximately 50% of Immunoprecipitated material), 4 µl was loaded for anti-HA blots (approximately 20% of Immunoprecipitated material) and 2 µl was loaded for anti-Ssn6 blots (approximately 10% of Immunoprecipitated material). For anti-HA co-IP blots, 10 µl was loaded for anti-Myc blots (approximately 50% of Immunoprecipitated material), 4 µl was loaded for anti-Ssn6 blots (approximately 20% of immunoprecipitated material) and 2 µl was loaded for anti-HA blots (approximately 10% of immunoprecipitated material).

### Western blots

Lysates were electrophoresed on 4–12% NuPAGE Bis-Tris gels with MOPS running buffer according to manufacturer’s instruction (Invitrogen). Separated proteins were transferred to a nitrocellulose membrane according to standard methods. Membranes were blocked with 5% NFDM (nonfat dry milk) in 1X TBS (20 mM Tris-Cl pH 7.5, 250 mM NaCl)/0.1% Tween. Following blocking, the membranes were incubated overnight in either a 1∶6000 dilution (in 1X TBS/0.1% Tween/5% NFDM) of rabbit anti-Ssn6 (Sharon Dent), a 1∶500 dilution (in 1X TBS/0.1% Tween/1% NFDM) of mouse anti-HA (Santa Cruz) or a 1∶2500 dilution (in 1X TBS/0.1% Tween/1% NFDM) of mouse anti-Myc (Upstate). The membranes were then washed three times in 1X TBS/0.1% Tween/1% NFDM and incubated in a 1∶15000 dilution (in 1X TBS/0.1% Tween/1% NFDM) of HRP conjugated donkey anti-rabbit IgG (anti-Ssn6 blots) or HRP conjugated donkey anti-mouse IgG (anti-HA and anti-Myc blots) (Amersham). Following washing, blots were developed by enhanced chemiluminescence (ECL) using an Amersham ECL Plus Detection Kit.

## Supporting Information

Figure S1 **Genes derepressed in a** ***tup1Δ*** **strain are bound by Tup1.** (**A**) Tup1 ChIP-chip data at single promoters are plotted versus derepression of the downstream genes in a *tup1Δ* strain [7]. (**B**) All genes were sorted into 10 bins depending on the degree to which they were derepressed in a *tup1Δ* strain [7]. The most derepressed genes are in the “90-100” bin the average Tup1, Ssn6, and Mock ChIP signal for unidirectional promoter genes in each bin is shown. Deciles
(TIF)

Figure S2 **Tup1 interacts with the known Tup1 recruiters Sut1, Nrg1, or Sko1.** This figure is a longer exposure for the same blot shown in Figure 4B. Strains carrying Myc-tagged predicted recruiters (Cin5, Phd1, Yap6, or Skn7), characterized recruiters (Sut1, Nrg1, or Sko1), or a protein which was not predicted to interact with Tup1 (Hap3) were immunoprecipitated with anti-Ssn6 antibodies, anti-HA antibody (to detect Tup1), and anti-MYC (to detect recruiter proteins).
(TIF)

Figure S3 **Characterization of DNAse I-treated Co-IP experiments.** Top, Genomic DNA isolated from the supernatant of Co-IP experiments in the presence or absence of DNAse I. Middle and bottom, To show digestion of the DNA, PCR was performed using genomic DNA prepared from the TOP panel as a template. The ability to amplify through small regions (∼400 bp) in the *RPS1A* gene (middle) and *Tup1-HA* tagged region (bottom) were examined.
(TIF)

Table S1 **Strains used in this study.**
(DOCX)

Dataset S1 **Tup1 bound sites with data shown in** **Figure 1** **.**
(XLSX)

Dataset S2 **Regulatory regions bound by each recruiter with Tup1 and Ssn6 occupancy.**
(XLSX)

Dataset S3 **All regulatory regions with regression model results compared to experimental results (data from** **Figure 5D** **).**
(XLSX)
