# Supplementary figures and images for: The Stress Response Factors Yap6, Cin5, Phd1, and Skn7 Direct Targeting of the Conserved Co-Repressor Tup1-Ssn6 in S. cerevisiae
Source: PLoS One. 2011 Apr 28;6(4):e19060. doi: 10.1371/journal.pone.0019060 (PMC3084262; doi:10.1371/journal.pone.0019060)

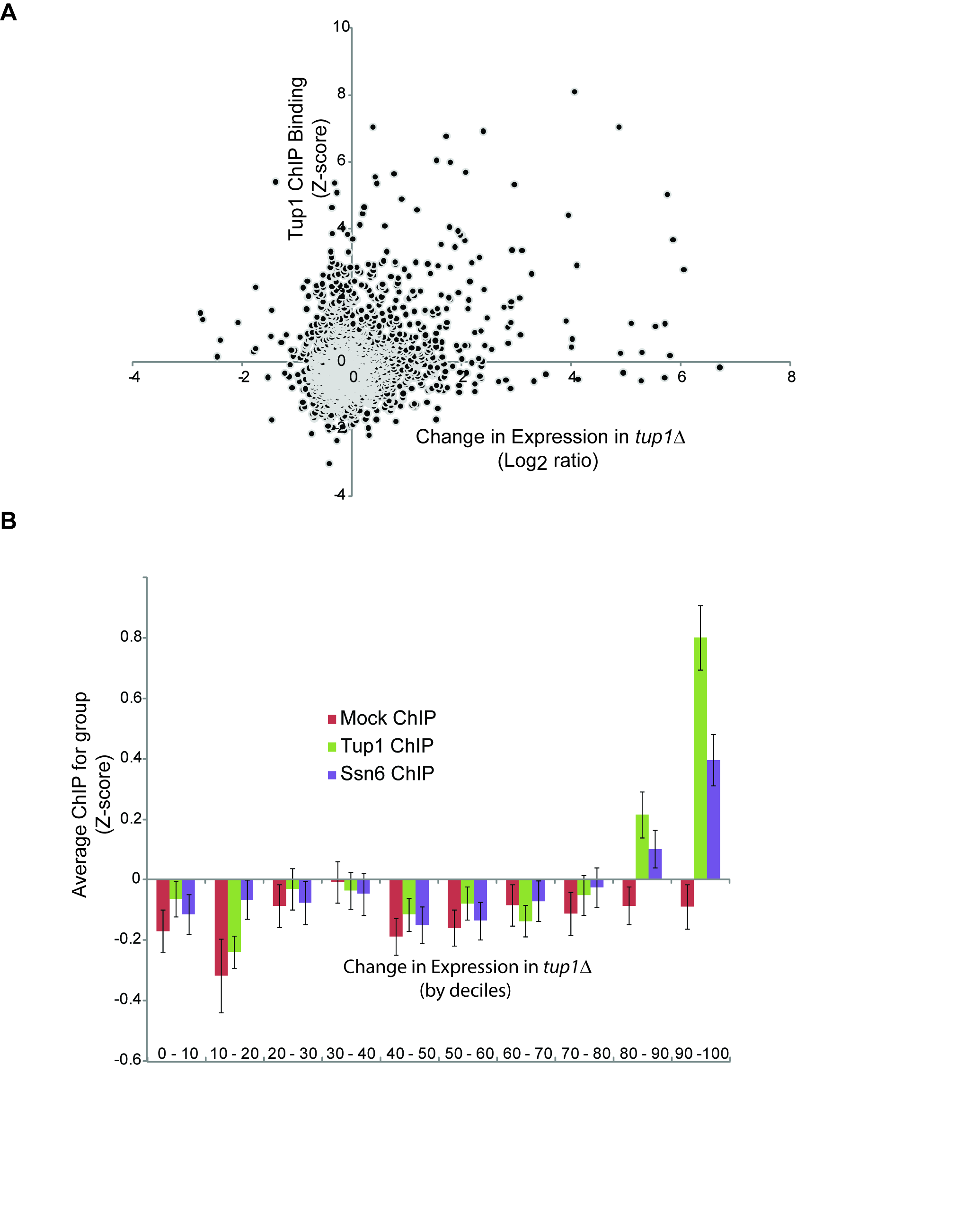

Supplement: Figure S1 — Genes derepressed in a tup1Δ strain are bound by Tup1. (A) Tup1 ChIP-chip data at single promoters are plotted versus derepression of the downstream genes in a tup1Δ strain [7]. (B) All genes were sorted into 10 bins depending on the degree to which they were derepressed in a tup1Δ strain [7]. The most derepressed genes are in the “90-100” bin the average Tup1, Ssn6, and Mock ChIP signal for unidirectional promoter genes in each bin is shown. Deciles (TIF) [file pone.0019060.s001.tif]

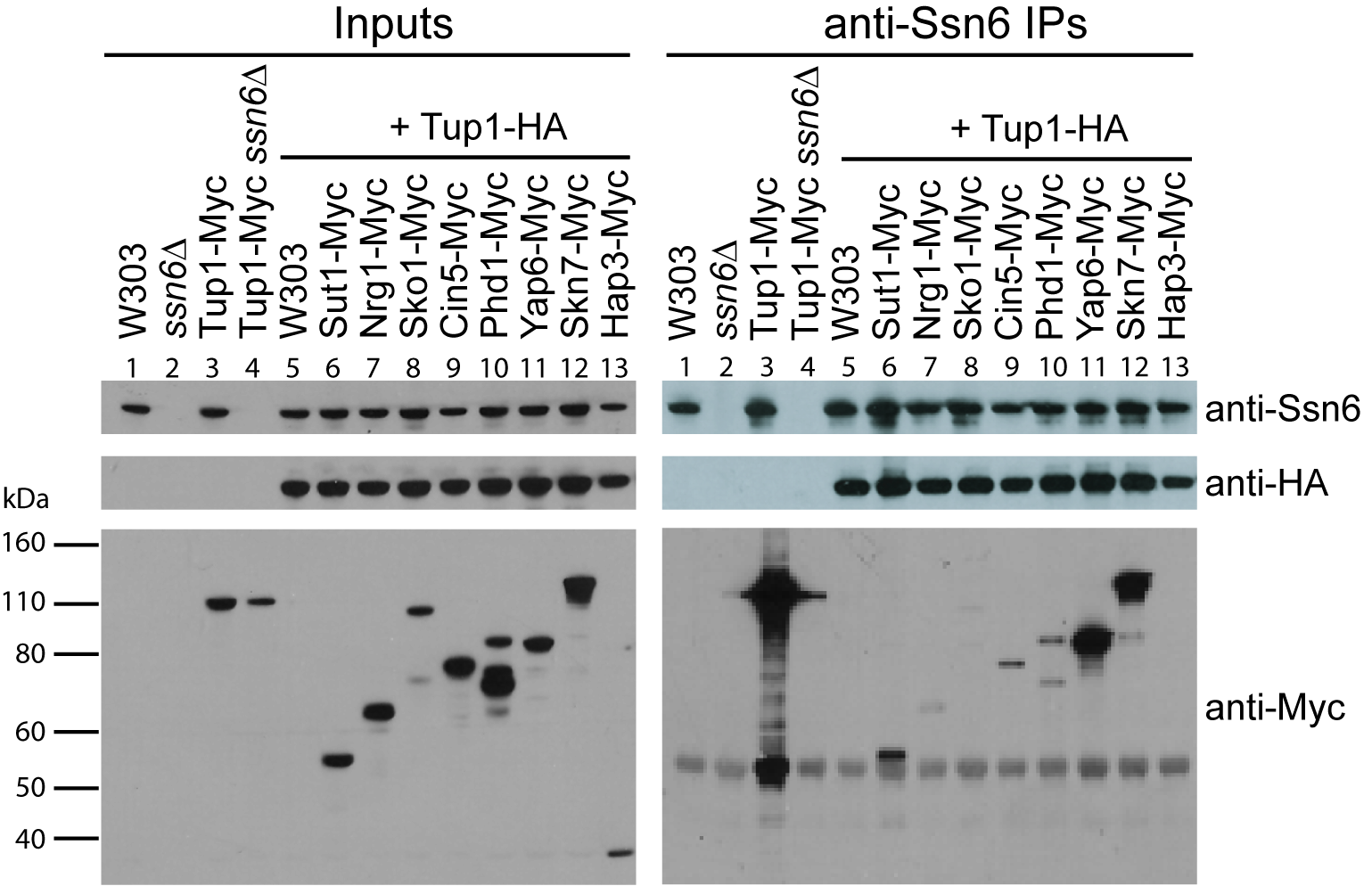

Supplement: Figure S2 — Tup1 interacts with the known Tup1 recruiters Sut1, Nrg1, or Sko1. This figure is a longer exposure for the same blot shown in Figure 4B. Strains carrying Myc-tagged predicted recruiters (Cin5, Phd1, Yap6, or Skn7), characterized recruiters (Sut1, Nrg1, or Sko1), or a protein which was not predicted to interact with Tup1 (Hap3) were immunoprecipitated with anti-Ssn6 antibodies, anti-HA antibody (to detect Tup1), and anti-MYC (to detect recruiter proteins). (TIF) [file pone.0019060.s002.tif]

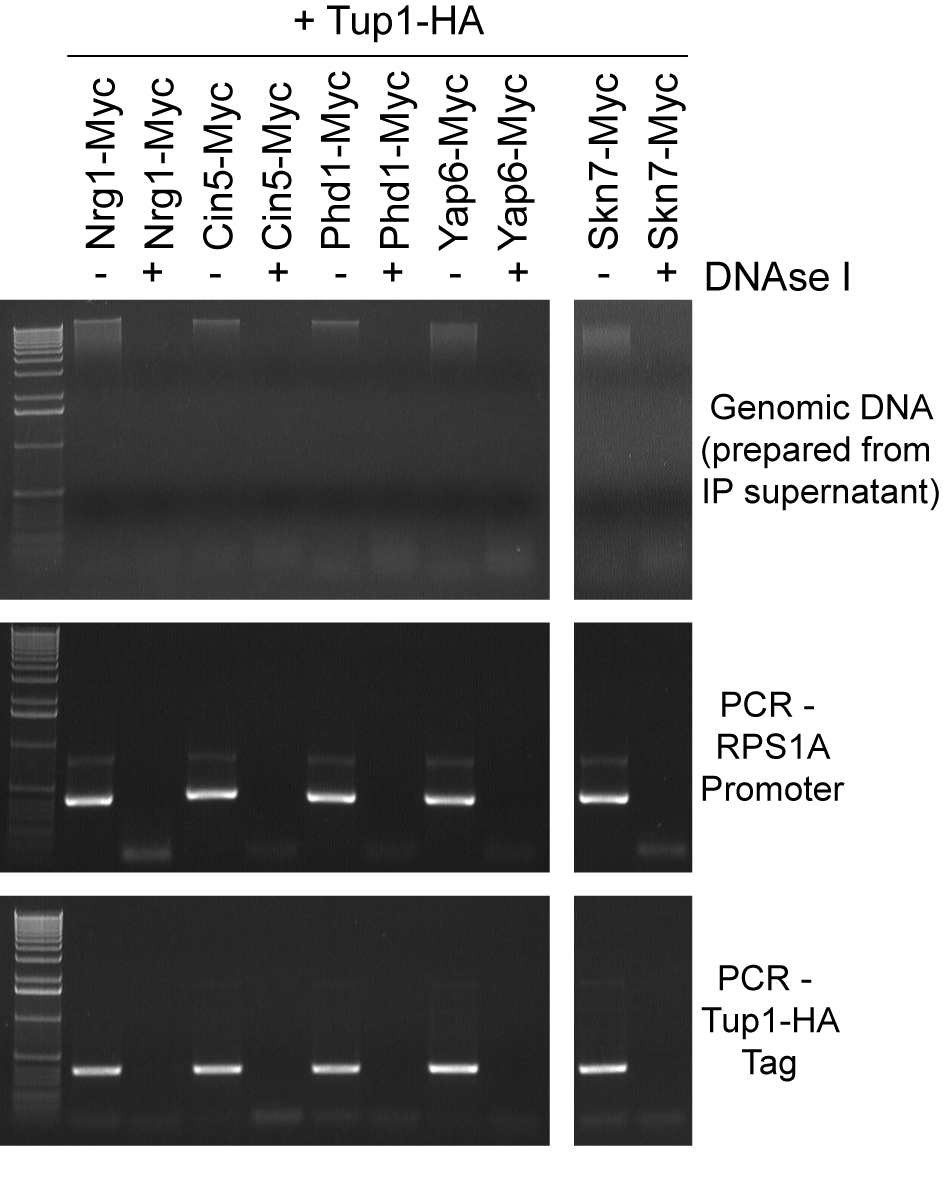

Supplement: Figure S3 — Characterization of DNAse I-treated Co-IP experiments. Top, Genomic DNA isolated from the supernatant of Co-IP experiments in the presence or absence of DNAse I. Middle and bottom, To show digestion of the DNA, PCR was performed using genomic DNA prepared from the TOP panel as a template. The ability to amplify through small regions (∼400 bp) in the RPS1A gene (middle) and Tup1-HA tagged region (bottom) were examined. (TIF) [file pone.0019060.s003.tif]
