# Supplementary material for: The Stress Response Factors Yap6, Cin5, Phd1, and Skn7 Direct Targeting of the Conserved Co-Repressor Tup1-Ssn6 in S. cerevisiae
Source: PLoS One. 2011 Apr 28;6(4):e19060. doi: 10.1371/journal.pone.0019060 (PMC3084262; doi:10.1371/journal.pone.0019060)
Supplement: Table S1 — Strains used in this study. (DOCX) [file pone.0019060.s004.docx]

**Table S1- Strains used in this study**

| **Strain** | **Genotype** | **Source** |
| --- | --- | --- |
| BY4741 | MATa his3Δ1 leu2Δ0 met15Δ0 ura3Δ0 |  |
| BY4742 | MATalpha his3Δ1 leu2Δ0 lys2Δ0 ura3Δ0 |  |
| SHy028 | MATa his3Δ1 leu2Δ0 met15Δ0 ura3Δ0 Tup1-TAP::HIS3 | Ghaemmaghami et al.[[1](#_ENREF_1)] |
| SHy048 | MATalpha his3Δ1 leu2Δ0 met15Δ0 ura3Δ0 Tup1-TAP::HIS3 | This Study |
| SHy061 | SHy028 but aft1Δ::KanMX6 | This Study |
| SHy062 | SHy028 but rfx1Δ::KanMX6 | This Study |
| SHy063 | SHy028 but mig1Δ::KanMX6 | This Study |
| SHy064 | SHy028 but nrg1Δ::KanMX6 | This Study |
| SHy065 | SHy028 but rox1Δ::KanMX6 | This Study |
| SHy066 | SHy028 but sko1Δ::KanMX6 | This Study |
| SHy067 | SHy028 but sut1Δ::KanMX6 | This Study |
| W303a | MATa leu2-3,112 trp1-1 can1-100 ura3-1 ade2-1 his3-11,15 phi+ |  |
| Z1451 | W303a but Cin5-myc::TRP1 | Harbison et al.[[2](#_ENREF_2)] |
| Z1334 | W303a but Phd1-myc::TRP1 | Harbison et al.[[2](#_ENREF_2)] |
| Z1365 | W303a but Skn7-myc::TRP1 | Harbison et al.[[2](#_ENREF_2)] |
| Z1541 | W303a but Yap6-myc::TRP1 | Harbison et al.[[2](#_ENREF_2)] |
| Z1533 | W303a but Sko1-myc::TRP1 | Harbison et al.[[2](#_ENREF_2)] |
| Z1744 | W303a but Sut1-myc::TRP1 | Harbison et al.[[2](#_ENREF_2)] |
| Z1535 | W303a but Nrg1-myc::TRP1 | Harbison et al.[[2](#_ENREF_2)] |
| Z1522 | W303a but Gts1-myc::TRP1 | Harbison et al.[[2](#_ENREF_2)] |
| Z1450 | W303a but Hap3-myc::TRP1 | Harbison et al.[[2](#_ENREF_2)] |
| SHy162 | W303a but Tup1-myc::hphNT1 | This Study |
| SHy163 | W303a but ssn6∆::KanMX6 | This Study |
| SHy164 | W303a but Tup1-myc::hphNT1 ssn6∆::KanMX6 | This Study |
| SHy165 | W303a but Tup1-HA::natNT2 | This Study |
| SHy166 | W303a but Sut1-myc::TRP1 Tup1-HA::natNT2 | This Study |
| SHy167 | W303a but Nrg1-myc::TRP1 Tup1-HA::natNT2 | This Study |
| SHy168 | W303a but Sko1-myc::TRP1 Tup1-HA::natNT2 | This Study |
| SHy169 | W303a but Cin5-myc::TRP1 Tup1-HA::natNT2 | This Study |
| SHy170 | W303a but Phd1-myc::TRP1 Tup1-HA::natNT2 | This Study |
| SHy171 | W303a but Yap6-myc::TRP1 Tup1-HA::natNT2 | This Study |
| SHy172 | W303a but Skn7-myc::TRP1 Tup1-HA::natNT2 | This Study |
| SHy174 | W303a but Hap3-myc::TRP1 Tup1-HA::natNT2 | This Study |
| SHy175 | W303a but Tup1-HA::natNT2 ssn6∆::KanMX6 | This Study |
| SHy176 | W303a but Sut1-myc::TRP1 Tup1-HA::natNT2 ssn6∆::KanMX6 | This Study |
| SHy177 | W303a but Phd1-myc::TRP1 Tup1-HA::natNT2 ssn6∆::KanMX6 | This Study |
| SHy178 | W303a but Yap6-myc::TRP1 Tup1-HA::natNT2 ssn6∆::KanMX6 | This Study |
| SHy179 | W303a but Skn7-myc::TRP1 Tup1-HA::natNT2 ssn6∆::KanMX6 | This Study |
| SHy183 | W303a but Nrg1-myc::TRP1 Tup1-HA::natNT2 ssn6∆::KanMX6 | This Study |
| SHy184 | W303a but Cin5-myc::TRP1 Tup1-HA::natNT2 ssn6∆::KanMX6 | This Study |
|  |  |  |

1. Ghaemmaghami S, Huh WK, Bower K, Howson RW, Belle A, et al. (2003) Global analysis of protein expression in yeast. Nature 425: 737-741.

2. Harbison CT, Gordon DB, Lee TI, Rinaldi NJ, Macisaac KD, et al. (2004) Transcriptional regulatory code of a eukaryotic genome. Nature 431: 99-104.
